# Lung microbial dysregulation and TNF inhibition contribute to worsened nontuberculous mycobacterial lung disease

**DOI:** 10.21203/rs.3.rs-8703262/v1

**Published:** 2026-02-05

**Authors:** Ethan Napier, Isaac Cinco, Ethan Stuart, Michael Davies, Caroline Leach, Evan Damron, Mahmut Gokmen, Amy Leestemaker-Palmer, Stephanie Nuss, Cody Bumgardner, Steven Kohama, Luiz Bermudez, Kevin Winthrop, Cristina Fuss, Eliot Spindel, Ilhem Messaoudi

**Affiliations:** University of Kentucky; University of Kentucky; University of Kentucky; Oregon National Primate Research Center; University of Kentucky; University of Kentucky; University of Kentucky; Oregon State University; Oregon State University; University of Kentucky; Oregon National Primate Research Center; Oregon State University; Oregon Health & Science University; Yale University; Oregon National Primate Research Center; University of Kentucky

**Keywords:** Nontuberculous mycobacteria, single cell RNA sequencing, microbiome, TNF, rhesus macaque

## Abstract

Nontuberculous mycobacteria (NTM) are ubiquitous bacteria that cause a spectrum of diseases, most notably pulmonary disease (NTMPD). The host factors contributing to the heightened susceptibility and severity of NTMPD in elderly individuals are poorly understood. Prior studies have reported increased incidence of NTMPD in individuals receiving immune modulatory biologics such as anti-TNF and JAK-STAT inhibitors. Moreover, we recently described that age-related changes in the lung microbiome, notably the loss of a main commensal *Tropheryma* species, may contribute to increased severity. Therefore, in this study we explore the hypothesis that TNF-inhibition and a disrupted lung microbiome are key factors that contribute to worse disease outcomes in older NTMPD patients. Young (4–6 years old) rhesus macaques were pretreated with nebulized amikacin and vancomycin to deplete the lung microbiome, pretreated with the TNF inhibitor Inflectra or left untreated. Animals were subsequently inoculated with *M. avium* subsp. *hominissuis* (MAH) in the right lung. Bacterial load, radiographic changes, immune responses, and microbiome composition were monitored longitudinally. Antibiotic-treated animals experienced significant dysbiosis including the depletion of *Tropheryma* from the lung microbiome. One antibiotic-treated animal developed and resolved cavitary disease after the lung microbiome returned to homeostasis. Inflectra-treated animals favored an acute-phase response that persisted up to 114 days after inoculation and one Inflectra-treated animal developed chronic granulomatous disease. No control animals showed granulomas. These data suggest that lung microbiome dysbiosis and TNF inhibition can increase susceptibility to NTM granulomatous disease.

## INTRODUCTION

Nontuberculous mycobacteria (NTM) are ubiquitous microbes that can infect human lungs via aerosolized particles^1,2^. Individuals with a compromised immune system or underlying lung conditions are at risk of developing NTM pulmonary disease (NTMPD) or disseminated NTM disease^3,4^. NTM disease first gained clinical relevance during the HIV/AIDS epidemic. These patients lacked a strong Th1 response due to the depletion of their CD4 + T cells, which allowed for disseminated NTM disease^5^. In contrast, most HIV-negative NTM cases are pulmonary. Risk factors for NTM infection can include pharmacological immunosuppression such as treatment with tumor necrosis factor-alpha (TNF) antagonists or underlying lung conditions including cystic fibrosis (CF), chronic obstructive pulmonary disease (COPD) and bronchiectasis. Age is another major risk factor, and it’s notable that most fatal NTM cases occur in people > 65^6,7^. Aging results in immunosenescence characterized by reduced circulating naïve T cells, increased memory T cells and chronic low-grade inflammation^8–11^. Additionally, aging dysregulates the lung microbiome, which can impact the prevalence and severity of respiratory diseases^12–14^.

An impaired Th1 immune response and a dysregulated lung microbiome may be underlying mechanisms for the increased susceptibility and severity of NTM disease in the aged population^15–17^. However, these mechanisms are poorly understood due to the limitations with rodent models of infection and human clinical studies. NTM infections in rodents can result in a disseminated rather than pulmonary disease^18^, and their specific-pathogen-free status prohibits translatable microbiome signatures^19–23^. Clinical studies face inherent challenges in sample acquisition and confounding variables like smoking history, diet, and comorbidities. We developed a rhesus macaque model that overcomes these limitations and recapitulates key hallmarks of human NTMPD including clinical and radiologic features, as well as immune response to infection^24^. Rhesus macaques are a widely accepted model for the impact of immunosenecence in respiratory infections^25,26^. Using this model, we previously demonstrated increased disease severity upon NTM infection with *M. avium* subsp. *hominissuis* strain 101 (MAH) in aged animals as measured by persistent radiographic findings consistent with bronchiectasis as well as presence of pulmonary lesions at necropsy, increased immune cell infiltration and activation by histopathology^13,27^.

These prior studies highlighted that while young animals generated a predominantly Th1 immune response (with high levels of IFNγ, IL-12, TNFα, and CD40L), aged animals generated predominantly an acute phase response indicated by high levels of IL6 and had prolonged elevation of the chemoattractant CCL2^13^. Moreover, the loss of an uncultured *Tropheryma* species in aged animals and the negative correlation between its abundance and the severity of radiographic findings suggested that the lung microbiome plays a protective against MAH disease^13,27^. These findings suggest that a Th1 response and *Tropheryma* abundance can modulate NTMPD severity. To test this hypothesis, we pretreated a cohort of rhesus macaques with either antibiotics to deplete the lung microbiome or the TNF antagonist Inflectra to inhibit Th1 immune responses. Infection was followed for 310 days with longitudinal CT imaging, microbiome profiling, immune cell characterization, histology and proteomic analysis. One Inflectra-treated animal developed pulmonary granulomatous disease, and one antibiotic-treated animal developed and resolved cavitary disease. Moreover, antibiotic- and Inflectra-treated animals showed significant dysregulation of immune mediators and immune cell infiltration compared to the control animals indicating that TNF signaling and dysbiosis of the lung microbiome can impact the outcome of MAH infection.

## RESULTS

### Antibiotic treatment and TNF blockade is associated with more severe disease presentation following MAH infection

**Figure 1:**
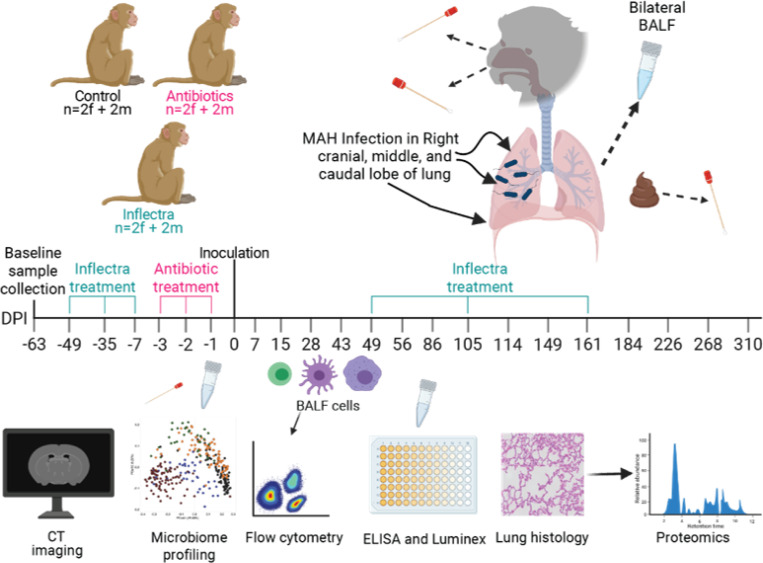
Experimental design. 12 young adult (5–10 years; 6 female and 6 male) rhesus macaques were segregated into 3 groups of 4: 1] Control, 2] antibiotic (Amikacin/vancomycin) administration, and 3] Inflectra administration. Animals were inoculated in the right cranial, middle, and caudal lobe with a bolus of 6.8×10^8^ CFU of MAH split between the three sites. Nasal, oral, fecal, and bilateral bronchoalveolar lavage fluid (BALF) samples were collected at the indicated days post inoculation (DPI). BALF was probed via microbiome profiling, flow cytometry, ELISA, and Luminex assay. Microbiome profiling was done on the nasal, oral, and fecal swabs. Computed tomography (CT) scans were acquired, and severity scores were determined 0, 12, 40, 83, 110, 145, 181, 222, 264, and 299 DPI. Lung histology was done at necropsy and proteomics data were acquired from the tissues.

Animals were randomly assigned to 3 groups with 4 animals each. The first group was left untreated (control group). The second group was nebulized with amikacin/vancomycin (antibiotic-treated group) 3-, 2-, and 1-day before infection. The third group was treated with Inflectra 7-, 5-, and 1-week before infection, as well as 49-, 105-, and 161-days post infection (DPI) to block TNF (Inflectra group). Animals in all three groups were inoculated with 6.8×10^8^ CFU MAH split between the right upper (cranial), middle, and lower (caudal) lobes Fig. 1. The right accessory lobe that macaques have was not inoculated with MAH.

**Figure 2:**
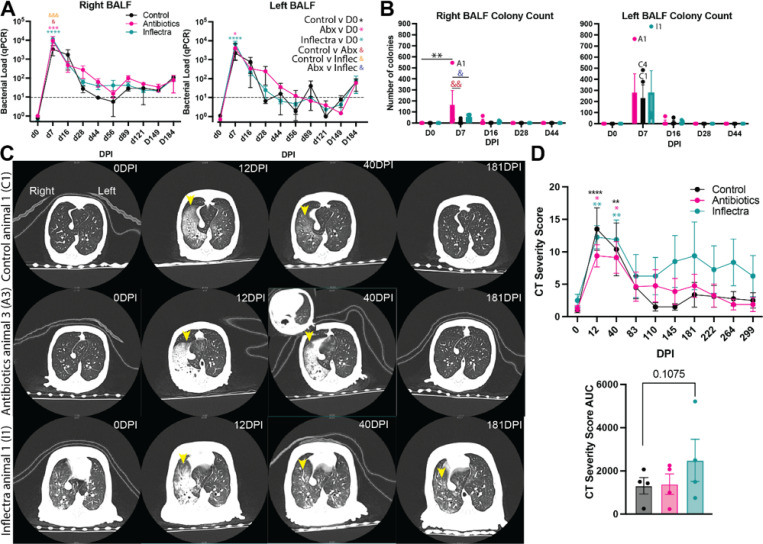
Antibiotic- and Inflectra-treated animals have increased bacterial load and worse disease outcomes. Bacterial load was measured in the BALF via **(A)** quantitative real-time PCR specific for the IS1311 sequence in *M. avium* and **(B)** plating on Lowenstein-Jensen agar plates to enumerate colony forming units (CFU) following 8 weeks of incubation. The dashed line represents the limit of detection. **(C)** Representative CT scans of antibiotic treated, control, and Inflectra treated animals before inoculation (0 DPI), at the height of inflammation (12 DPI) or of cavity formation (40 DPI), and during resolution (181 DPI). Yellow arrow heads point out inflammation. **(D)** CT scans were analyzed by a blinded cardiothoracic radiologist and reported as severity scores at the indicated days post inoculation (top) and as an area under the curve analysis (bottom). Data from the control group (C1-C4) are plotted in black, from the antibiotic group (A1-A4) are plotted in pink, and from the Inflectra-treated group (I1-I4) are plotted in teal. In panels A-C, data are average ± SEM. *p<0.05, **p<0.01, ***p<0.001, ****p<0.0001.

We measured bacterial load in the right and left bronchoalveolar lavage fluid (BALF) of all animals with real-time quantitative PCR (RT-qPCR) and by plating to solid growth medium. In line with previous studies^13,27^, both methods revealed peak bacterial burden 7 DPI that became undetectable at 44 DPI in both the left and right BALF (Fig. 2A-B). RT-qPCR showed a modest, but significantly larger peak bacterial load in antibiotic- and Inflectra-treated animals compared to control animals in the right BAL (Fig. 2A). This was also observed by colony counts in antibiotic-treated animals (Fig. 2B). Interestingly, no differences in bacterial load or colony numbers between all the groups were detected in the left BAL (Fig. 2B).

Computed tomography (CT) was performed throughout the infection and scored by a blinded cardiothoracic radiologist based on radiographic changes such as bronchiectasis, ground glass, and tree-in-bud opacities (Fig. 2C-D). Consistent with the height of bacterial burden, CT severity scores peaked at 12 DPI in all 3 groups (Fig. 2C-D, S1A-C). As observed in our prior studies, overt radiographic findings in control animals were resolved by 83 DPI and remained unremarkable for the remainder of the study (Fig. 2C). One antibiotic-treated animal 3 (A3; female) developed a 16 × 20 mm cavity at 40 DPI, that expanded to 1.6 × 2.2 cm at 83 DPI and decreased to 4 × 5 mm by 299 DPI (Fig. 2C). The remaining 3 antibiotic-treated animals showed comparable radiographic changes as the control group **(Figure S1B)**. One inflectra-treated animal 1 (I1; male) showed persistent inflammatory changes, while the remaining 3 animals showed a comparable presentation as the controls (Fig. 2C, S1C-D). Gross pathology findings showed areas of pulmonary consolidation in A3, A4, and I1 **(Figure S2).**

**Figure 3:**
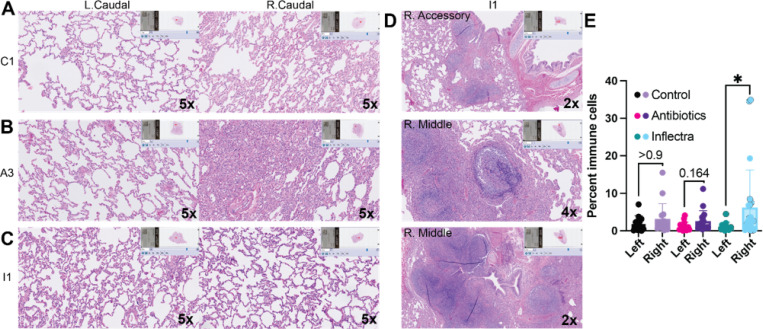
Antibiotic-treated animal A3 exhibits pulmonary consolidation and Inflectra-treated animal I1 exhibits granulomatous disease. H&E stains at the indicated magnifications of the left and right caudal lobes of **(A)**control animal 1, **(B)** antibiotic-treated animal 3, and **(C)** Inflectra-treated animal 1. **(D)** H&E stains at the indicated magnifications of the right accessory and middle lobes of animal I1. **(E)** Percent immune cells from the right and left lungs of antibiotic treated, control, and Inflectra-treated animals. Circles with black borders are from animal I1.

Hemotoxylin and eosin (H&E) staining revealed that lungs of control animals were unremarkable at necropsy (Fig. 3A, S3). Lung sections from antibiotic-treated animals showed inflammatory infiltrates in the right caudal lobe of antibiotic-treated animal A3, which had developed cavitary disease at day 40 (Fig. 3B, S3). Finally, the right accessory and right middle lobes of animal I1, which showed persistent inflammatory changes by CT, showed granulomatous disease (Fig. 3D). Quantification of immune infiltrates revealed a significant increase in the percentage of immune cells in the right compared to left lungs of Inflectra-treated animals driven by animal I1 (Fig. 3E).

### Antibiotic or Inflectra treatment results in distinct inflammatory signatures and immune cell composition in the lung following MAH infection.

**Figure 4:**
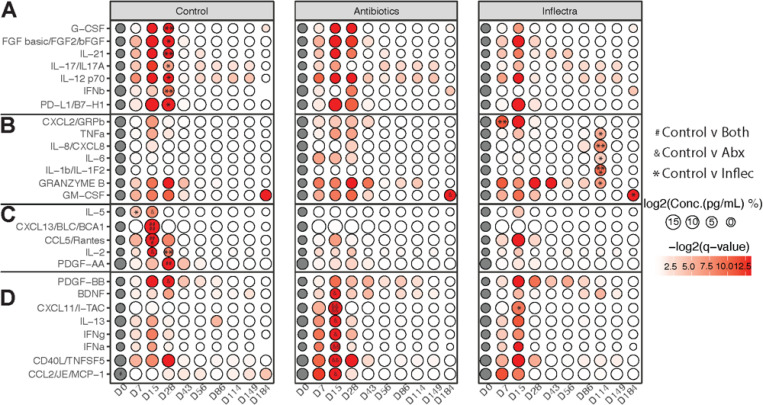
Antibiotic-treated animals have an unregulated Th1 response while Inflectra-treated animals favor an acute-phase response. **(A-D)** Bubble plot of the differentially abundant immune mediators in BALF supernatants determined by Luminex assays at the indicated timepoints in the right lungs of the control, antibiotic-treated, and Inflectra-treated groups. The size of the bubble represents the concentration of the immune mediator while color represents the significance of the change. # indicates a significant difference between the control group compared to both the antibiotic- and Inflectra-treated groups. & indicates a significant difference between the control and antibiotic groups. * indicates a significant difference between the control and Inflectra groups *p < 0.05, **p < 0.01, ***p < 0.001, ****p < 0.0001.

We next longitudinally measured levels of immune mediators in the BALF by Luminex (Fig. 4). Growth factors G-CSF and FGF basic as well as pro-inflammatory mediators IL-21, IL-17, IL-12, IFNβ, and anti-inflammatory PD-L1 were higher in the control group 28 DPI compared to the Inflectra-treated group (Fig. 4A). Levels of chemoattractants IL-5 (eosinophil), CXCL13 (B cell), CCL5 (T cells and monocytes) as well as IL-2 (T cell modulator) and PDGF-AA (cell division) were increased in controls compared to both Inflectra- and antibiotic-treated animals 7–28 DPI (Fig. 4C). Finally, PDGF-BB (vascular remolding and cell proliferation) levels were increased in the control versus the antibiotic-treated group at 28 DPI.

On the other hand, the Inflectra-treated group had higher levels of CXCL2 (leukocyte chemoattractant) at 7 DPI (Fig. 4B), as well as higher levels of TNF (Th1 response), IL-8 (neutrophil chemoattractant), IL-6 (acute phase response), IL-1β (induces IL-6 and TNF), and Granzyme B (cytotoxic T lymphocyte protease) at 114 DPI compared to control animals (Fig. 4A). The granulocyte growth factor GM-CSF level was increased in BALF of both Inflectra- and antibiotic-treated animals 184 DPI (Fig. 4B). The antibiotic-treated group exhibited higher levels of BDNF (growth factor), CXCL11 (T cell and NK cell chemoattraction), IL-13 (mucus secretion), IFNγ, IFNα, CD40L (co-stimulatory factor), and CCL2 (monocyte/macrophage chemoattractant) 15 DPI compared to controls (Fig. 4D). These data indicate an increased innate immune response in the Inflectra-treated group (increased IL-8 and IL-6) while the antibiotic-treated group exhibited a stronger adaptive immune (CXCL11, CD40L) and Th1 (increased IFNγ) response.

**Figure 5:**
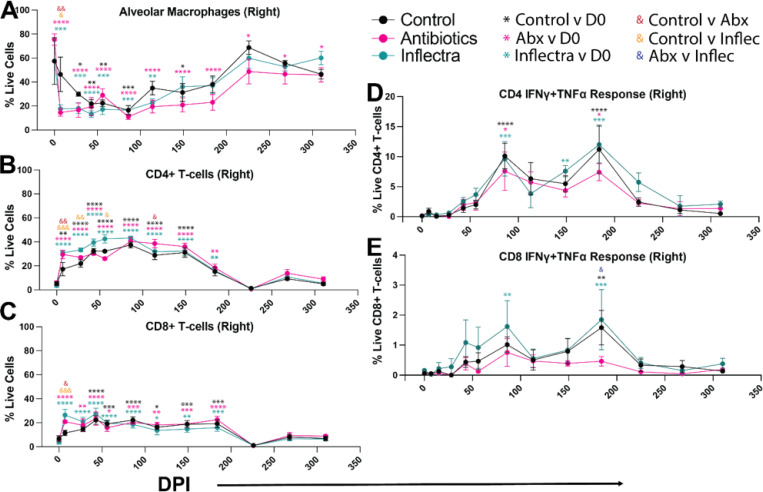
Inflectra-treated animals have modestly more T cell influx. BALF samples were collected longitudinally from the right lungs at the indicated DPI and flow cytometry was performed to enumerate the relative frequency within live cells of **(A)** alveolar macrophages; **(B)** CD4+ T-cells, **(C)**CD8+ T cells, **(D)** MAH-specific IFNγ+ TNF+ CD4 T-cells, and **(E)** MAH-specific IFNγ+ TNF+ CD8 T-cells. The MAH-specific CD4 and CD8 T-cell response was determined by intracellular cytokine staining following stimulation with 1 mg/mL MAH lysate. Data are average ± SEM. Two-way ANOVA statistical tests were performed to evaluate within group changes compared to 0 DPI (denoted by asterisks) and between group changes at each timepoint (denoted by ampersands). *p < 0.05, **p < 0.01, ***p < 0.001, ****p < 0.0001.

Macrophage and T cell frequencies and MAH-specific responses were longitudinally evaluated by flow cytometry. Right and left BALF in all animals was composed of ~ 60% alveolar macrophages (AMs) at the time of infection (Fig. 5A, S4A). AM relative abundance decreased dramatically at 7 DPI in the right lungs (site of inoculation) of antibiotic- and Inflectra-treated animals, and 28DPI in controls, returning to pre-infection levels after 149 DPI in controls and Inflectra-treated animals but not antibiotic-treated animals. Loss of AM in the left BAL was only evident in the antibiotic- and Inflectra-treated groups returning to pre-infection levels after 86 DPI **(Figure S4A)**.

The loss of AMs starting at 7 DPI was concurrent with an influx of CD4 + and CD8 + T cells (Fig. 5B-C, S4B-C). The antibiotic- and Inflectra-treated groups showed an earlier CD4 and CD8 T cell influx in the right lung than controls (7 DPI), but T cell levels in all groups were elevated between 28 DPI and 226 DPI (Fig. 5B-C). We then investigated frequency of antigen-specific T cells using intracellular cytokine staining following stimulation with MAH lysate. Interestingly, MAH-specific CD4 T cell responses were only evident starting 86 through 184 DPI reaching statistical significance only sporadically (Fig. 5D). MAH-specific CD8 T cells were detected in the right lung of Inflectra-treated animals 86 and 184 DPI and in control animals at 184 DPI but not in the antibiotic-treated group (Fig. 5E).

Within the left lungs, CD4 T cell levels were elevated in the inflectra-treated group compared to controls 28 and 86 DPI (**Figure S4B**). CD8 T cells were higher in the antibiotic-treated group 28–43 DPI and in the Inflectra-treated group 28–86 DPI but were not significantly increased in the control animals (**Figure S4C**). CD4 T cell responses in the left lung were detected with similar kinetics as the right lung except for the control animals at 86 DPI likely due to the large standard deviation within the control group (**Figure S4D**). The Inflectra-treated group exhibited more CD4 and CD8 T cells in the left lung 184 DPI compared to control and antibiotic groups (**Figure S4D-E**). MAH antigen-responsive CD8 T cells were only detected in left lungs of Inflectra-treated animals 86 and 184 DPI (**Figure S4E**).

We measured MAH-specific IgG antibody titers performed in plasma and BALF supernatants. Circulating MAH-specific IgG antibodies were slightly higher in plasma from control animals compare to the antibiotic-and Inflectra-treated groups (**Figure S4F**). No significant differences in IgG titers were observed in the right BALF (**Figure S4G**). Collectively, these data suggest that antibiotic- and inflectra-treated animals have more T cell influx during their immune response to MAH.

### Lung microbiome dysbiosis is induced by antibiotic and Inflectra treatment as well as infection

**Figure 6:**
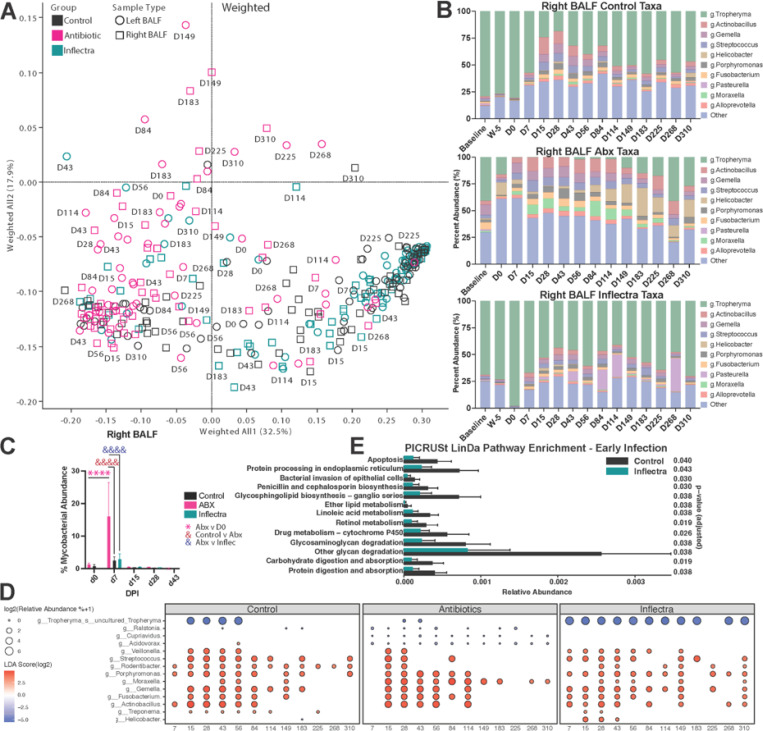
Lung *Tropheryma*abundance is decreased by antibiotic treatment but increased by Inflectra treatment. **(A)** The weighted principal coordinate analysis (PCoA) plot of the left and right BALF at the indicated DPI. **(B)** Bar plots displaying the dominant phyla in the right BALF and their percent relative abundances at the indicated timepoints. Baseline is the timepoint before any treatments were ever done. **(C)**Percent *Mycobacterium* abundance as measured by 16S rRNA sequencing in the right BAL. **(D)** Bubble plot derived from LEfSe analysis of the right BALF microbiome profiling. The size of each bubble represents the percent relative abundance and color represents the linear discriminant analysis (LDA) score. **(E)** Phylogenetic investigation of communities by reconstruction of unobserved states (PICRUSt) using linear models for differential abundance analysis (LinDA) at early-infection timepoints (7–86 DPI) between control and Inflectra-treated animals. Pathway enrichments were filtered based on a log2 fold change > 1.5 and adjusted p-value < 0.05.

To profile the BALF, oral, nasal and fecal microbiomes, we performed 16S rRNA sequencing. Weighted and unweighted principal coordinate analysis (PCoA) of control animals revealed distinct separation with each sample type falling into unique quadrants (Fig. 6A, S5A). The Inflectra-treated samples largely resembled controls, but the BALF samples from antibiotic-treated animals were distinct (Fig. 6A). We investigated the top 10 most abundant genera in the right MAH-infected BALF of each experimental group (Fig. 6B). As we previously observed^13,27^, *Tropheryma* dominated the lung microbiome of the control and Inflectra-treated groups before infection and were reduced following infection (Fig. 6B, S5B). Animals in the antibiotic-treated group that received a dose of amikacin and vancomycin 3 days before infection had reduced *Tropheryma* 0 DPI compared to the control and Inflectra groups (Fig. 6B). Interestingly, antibiotic-treated animal A3 had a relative abundance of *Tropheryma* of 0.39 at baseline (before antibiotic treatment and MAH infection). A4, which showed signs of lung consolidation, had a *Tropheryma* relative abundance of 6.55%, while A1 and A2 showed minimal radiographic changes had relative abundances of 99.29 and 56.58, respectively. These observations are in line with our prior reports that suggested that relative abundance of *Tropheryma* negatively correlates with disease severity^13,27^. The overall microbial load in the right lung of control and antibiotic-treated animals did not drastically change due to antibiotic treatment or infection **(Figure S5C)**. In contrast, the microbial load of Inflectra-treated animals was significantly increased by 85 and 113 DPI **(Figure S5C)**. The antibiotic-treated group exhibited significant changes in the overall diversity of their right lung microbiome 86 DPI and in the left lung 15, 28, 86, and 149 DPI **(Figure S5D)**.

Linear discriminant analysis effect size (LEfSe) was performed on the right BALF to investigate the longitudinal changes of specific microbes following MAH inoculation compared to 0 DPI within each group (Fig. 6C-D). In line with the qPCR and culture data, reads mapping to *Mycobacteria* species were more abundant at 7 DPI in the antibiotic-treated animals compared to the Inflectra-treated and control animals (Fig. 6C). *Tropheryma* were drastically reduced in all three groups after MAH infection (Fig. 6D). Levels of *Tropheryma* recovered in control animals and Inflectra-treated animals, but this recovery was significantly disrupted in antibiotics-treated animals. Relative abundance of several nasal and oral commensals (*Veillonella, Streptococcus, Rodentibacter, Porphyromonas, Moraxella, Gemella, Fusobacterium, Actinobacillus*) was increased throughout the study in all groups while other upper respiratory tract microbes *Ralstonia*, *Cupriavidus*, and *Acidovorax* were reduced only in antibiotic-treated animals (Fig. 6D). DNA associated with the gut commensal *Treponema* appeared in the BALF of the control and Inflectra-treated groups at 15–28 DPI while that associated with the gut commensal *Helicobacter* was detected 15–43 DPI in Inflectra-treated animals (Fig. 6D).

We subjected the BALF 16S amplicon sequencing data to phylogenetic investigation of communities by reconstruction of unobserved states (PICRUSt) to infer the functional capabilities of the lung microbiome^28^ (Fig. 6E). We performed comparisons at pre-infection, early-infection (7–84 DPI), and late-infection (114–310 DPI) between control and antibiotic-treated groups as well as between control and Inflectra-treated groups. Only control versus Inflectra-treated groups at early-infection showed significant differences (log2 fold change > 1.5 and adjusted p-value < 0.05) when using linear models for differential abundance analysis (LinDA). Specifically, enrichment to pathways associated with protein processing, bacterial invasion of epithelial cells, biosynthesis of penicillin, cephalosporin, and glycosphingolipid as well as metabolism of ether lipid, linoleic acid, and retinol was predicted to be decreased in the Inflectra-treated group (Fig. 6E). These data suggest that Inflectra treatment leads to suppression of several critical pathways (e.g. biosynthesis, metabolism, and absorption) following MAH infection.

The nasal microbiome of antibiotic-treated animals was initially more diverse than the other groups at 7 DPI **(Figure S6A)**. *Psychrobacter* in the nasal community was most affected by antibiotic administration but recovered to baseline levels by 7 DPI **(Figure S6A)**. The oral microbiome of the antibiotic group exhibited more diversity 28, 43, 114, and 184–268 DPI **(Figure S6C)**. The fecal microbiome diversity of the antibiotic group was lower than that of the Inflectra-treated group 0 DPI **(Figure S6C)**. Fecal diversity was increased in all groups 15–56 and 226 DPI and returned to baseline by 310 DPI **(Figure S6C)**.

### Inflammatory changes are evident in lesioned tissue from antibiotic- and Inflectra-treated animals

**Figure 7:**
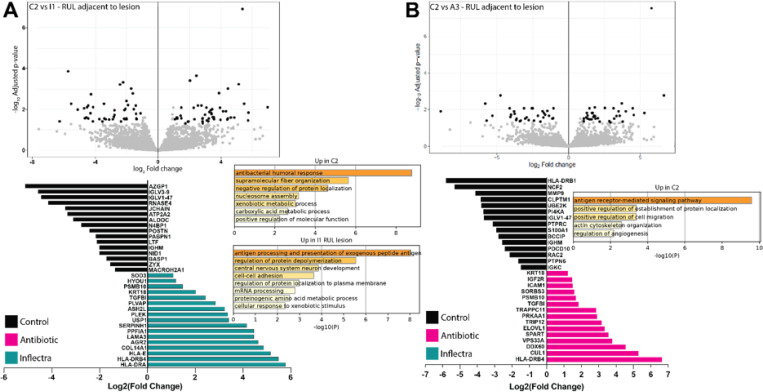
Antibiotic- and Inflectra-treated animals lack humoral immune responses compared to control animals. Differentially abundant proteins and Gene Ontology (GO) enrichment bar plots in the left lower lung tissue of **(A)** animal C2 compared to the right upper lobe that was near a lesion of animal I1 and **(B)** animal C2 compared to the right upper lobe that was near a lesion in A3.

We performed proteomic data acquisition and analysis on healthy lung tissue from the left lower lobe of control C2, the right upper lobe of Inflectra-treated I1 that was adjacent to a lesion, the right middle lobe of Inflectra-treated I2 near a site of inflammation on CT, the right upper lobe of antibiotic-treated A3 that was adjacent to a lesion, and the right middle lobe of A3 (Fig. 7A-B, S7A-B). Comparison of lung tissue from C2 to the right upper lung of I1 that was adjacent to a lesion showed increased expression of 43 proteins in C2 relative to I1 and 50 proteins overexpressed in I1 compared to animal C2 (Fig. 7A). Proteins more abundant in C2 were indicative of an antibacterial humoral response (IGLV3–9, IGLV1–47, JCHAIN, IGHM) as well as lung tissue remodeling (ALDOC, POSTN, NID1, ZYX). On the other hand, the proteome of I1 was enriched in antigen presentation (HLA-E, HLA-DRB4, HLA-DRA), tissue remodeling (KRT18, COL14A1, TGFBI, PLVAP, and LAMA3), protein depolymerization (PSMB10), reactive oxygen species (SOD3), and hypoxia (HYOU1) (Fig. 7A). Similarly, the sample from I2 was enriched in proteins related to inflammation and antigen presentation (IGSF8, IGHG1, HLA-DRA, HLA-DRB4, TNFAIP2, IGLV3–16, S100A8, GBP4, S100A9, MRC1, S100A14) and wound response (EPS8, CLDN5, CADM1, PDGFRB, ITGAV, CD9, MRC1) **(Figure S7A)**.

Comparison of lung tissue from animal C2 to the lesion in animal A3 revealed 32 overexpressed proteins in C2 and 35 overexpressed in A3 (Fig. 7B). The control lung was enriched to antigen-receptor mediated signaling (HLA-DRB1, UBE2K, PI4KA, PTPRC, PTPN6) and the humoral response (IGLV1–47, IGHM, IGKC). Abundant proteins in A3 were involved in tissue organization and development (KRT18, IGF2R, TGFBI, SPART, CUL1), cellular adhesion (ICAM1, SORBS3), autophagy (PRKAA1, VPS33A), and immune responses (PSMB10, DDX60, HLA-DRB4) (Fig. 7B). The tissue from the right middle lobe of animal A3 showed higher abundances of proteins important for antigen presentation (HLA-DRB4, HLA-DRA) and tissue repair (SERPINH1, ITGA6, IGF2R, ITGAV, TGFBI, PDGFRB) **(Figure S7B)**..

## DISCUSSION

Clinical and genome-wide association studies (GWAS) indicate that effective immunity against NTM requires robust Th1 T cell responses^15^. Mutations in key Th1 pathway genes such as IL-12 subunits, STAT1, and interferon stimulated genes are linked to increased susceptibility to NTMPD in children^29–32^. Therapies that block TNFα further elevate this risk^16,17^. Consistent with this, peripheral blood mononuclear cells from individuals with NTMPD produce less IFNγ and TNFα than those from healthy controls^17,33^. Additionally, a dysbiotic respiratory microbiome has been implicated as a host factor in NTMPD, as reflected by distinct community profiles in healthy versus NTM-infected lungs^34–36^. Indeed, in a cohort of women with a history of breast cancer, the sputum from women with NTMPD compared to those without show reduced abundance of *Leptotrichia*, *Streptococcus*, and *Veillonella*^35^. In addition, BALF from patients with NTMPD is enriched in *Ralstonia*, *Clavibacter*, and *Enterobacter* relative to individuals with non-CF bronchiectasis^36^. Collectively, this work suggests that Th1 immune responses and the lung microbiome are important factors during NTMPD. Therefore, we examined the role of the TNF immune signaling and the lung microbiome on pulmonary MAH infection in rhesus macaques. One animal whose TNF signaling was blocked via Inflectra treatment developed pulmonary granulomatous disease, and one animal with a depleted lung microbiome developed cavitary disease. These two cases support the importance of Th1 responses and a healthy microbiome to the anti-NTM response.

In all groups, bacterial load was highest at 7 DPI followed by a rapid clearance of MAH. Animals treated with antibiotics or Inflectra showed higher level of MAH at 7 DPI as quantified through either culture or qPCR. These results suggest that these treatments resulted in either greater bacterial colonization or decreased ability of the host to control early infection resulting in higher rates of MAH replication. Disease outcomes for animals I1 (severe inflammation and granulomatous disease) and A3/A4 (cavitary disease, consolidation), suggest that the host immune response is impaired in animals with disrupted TNF signaling or dysbiotic microbiomes.

Several important differences in immune mediator production between the control and treated cohorts were noted. Early in infection, control animals had significantly higher G-CSF, IL-21, IL-17, IL-12, IFN-β and PD-L1 than the Inflectra-treated animals. Increased levels of pro-repair growth factors (G-CSF)^37,38^, anti-inflammatory PD-L1^39^, as well as crucial cytokines for initiating type 1 immune programs (IL-12)^40^ and lung immunity (IL-17)^41^ suggest a balanced immune response to infection in the control group. On the other hand, the Inflectra-treated animals had a brief spike of TNF production in the right lungs compared to the control animals at 114 DPI, which may suggest a compensatory mechanism to the Inflectra treatment received at 105 DPI^42^. Inflectra-treated animals also overexpressed IL-6, IL-8, IL-1β at this singular 114 DPI timepoint, suggesting that these animals relied on a non-Th1 immune response to MAH^43^. Proteomic analysis indicated upregulation of antigen processing and presentation pathways in Inflectra-treated I1, in contrast to the humoral response signatures seen in control C2. This suggests that in the Inflectra-treated animals, the innate immune response is stimulated but is unable to effectively induce adaptive immunity due to TNF inhibition^44,45^.

Antibiotic-treated animals have increased levels of CXCL11, IFNα, IFNγ and CD40L compared to control animals at 15 DPI, suggesting a stronger type 1 immune response^43^. Despite the increased cytokines in the BALF of antibiotic-treated animals at 15 DPI, antigen presentation (HLA-DRB4, B2M) was the only proteomic evidence of immune involvement in the lung tissue of animal A3 at necropsy (310 DPI). Instead, several tissue repair and remodeling proteins such as IGF2R, TGFBI, and PDGFRB that could contribute to the cavitary resolution were increased^46^.

Previous findings indicated that a dysbiotic respiratory microbiome contributes to the development of NTMPD. We posit that the differing immune states observed at 15 DPI in the antibiotic-treated animals may be due to the depletion of *Tropheryma* since *Tropheryma whipplei* has been reported to reprogram macrophages to an M-2 like state^47,48^. Antibiotic-treated animals had a significantly lower *Tropheryma* abundance at the onset of infection that was further reduced after MAH infection and did not rebound to the same extent as seen in the control animals. Notably, antibiotic-treated animal A3 that developed cavitary disease had the lowest relative abundance of *Tropheryma* at baseline (before antibiotic treatment and MAH infection) compared to the other antibiotic-treated animals. In control animals, the initial *Tropheryma* abundance, depletion after MAH infection, and recovery by the end of the study was consistent with our previous work^13^.

In this study, we tested the hypothesis that the lung microbiome and TNF immune responses were responsible for effectively controlling MAH pulmonary infection. Antibiotic-treatment and Inflectra-treatment showed trends of enhanced pathology. This aligns with our prior work where aged animals uniformly showed more severe clinical outcomes following MAH infection^13,27^. We note that the low sample size of four macaques in each group is a limitation. Additionally, we only impaired young animals’ ability to control MAH infection by inhibiting TNF signaling or the lung microbiome alone. Together, these observations indicate that reduced Th1 or lung microbiome dysbiosis alone are not sufficient to recapitulate age-mediated increased susceptibility to NTM infection. This further supports the notion that increased susceptibility is multifactorial and that changes in epithelial cells and mucociliary escalator are likely critical components. It is possible that combining the two treatments in future studies would better replicate the disease outcome observed in aged macaques.

## MATERIALS AND METHODS

### Ethics statement:

Animal work was performed in accordance with the recommendations described in the Guide for the Care and Use of Laboratory Animals of the National Institute of Health, the Office of Animal Welfare and the Animal Welfare Act, United States Department of Agriculture. The studies were approved by the Institutional Animal Care and Use Committees (IACUC) at the Oregon National Primate Research Center.

### Cohort description, animal infection, and sample collection:

Twelve young (5–10 years; 6 females, 6 males) colony-bred Indian-origin rhesus macaques (*Macaca mulatta*) were separated into three groups: control, pre-treatment with 15 mg/kg amikacin and 4 mg/kg vancomycin 3, 2, and 1 day prior to infection (antibiotic-treated), as well as pre-treatment with 5 mg/kg Inflectra 7, 5 and 1 week prior to infection followed by treatment with Inflectra 49, 105, and 161 DPI (inflectra-treated) (**Table S1**). Following pre-treatment, animals were intrabronchially inoculated in the right upper, middle, and caudal lobe with a total of 6.8×10^8^ CFU of *M. avium* subsp. *hominissuis* strain 101 (MAH) divided equally between the 3 sites^27^. Thoracic CT, bronchoalveolar lavage fluid (BALF), oral, nasal, and fecal swabs were collected throughout the study (Fig. 1A).

### CT severity scoring system:

CT scans were evaluated by a blinded radiologist and assigned a severity score based on appearance of ground glass, mucus, volume loss, lymphadenopathy, detection and severity of bronchiectasis, presence and size of cavitations, scarring, and tree in bud opacity with a score of [1] being minimal findings to [6] being severe.

### Bacterial culture and load:

Bacterial load was determined by [1] plating serial dilutions of the BALF supernatant onto Lowenstein-Jensen agar plates and enumerating the number of colony-forming units (CFU) in after 8 weeks of incubation at 37°C/5% CO_2_ and [2] performing real-time quantitative PCR (qPCR). DNA was extracted from BALF cells using the DNeasy PowerSoil Pro Kit (Qiagen, Germantown, MD) according to the manufacturer’s instructions. Bacterial burden of MAH at 0, 7, 16, 28, and 44 DPI was determined by qPCR using SsoAdvanced Universal SYBR Green Supermix (Bio-Rad, Hercules, CA) and primers specific for the IS1311 insertion sequence found in *M. avium*^49^. Each run was initiated at 50°C for 2 min, then 95°C for 10 min, followed by 40 cycles at 95°C for 15 sec then 60°C for 1 min using a QuantStudio 3 Real-Time PCR System (Thermo Fisher Scientific). Extracted MAH DNA was used as the quantification standard.

### Flow cytometry:

1 × 10^6^ BALF cells were incubated with Ghost Dye Violet 510 (Tonbo Biosciences, San Diego, CA), then surface stained with antibodies against CD20, CD27, IgD, CD4 (Biolegend, San Diego, CA), CCR7 (BD Biosciences, Franklin Lakes, NJ), CD8b (Beckman Coulter, Brea, CA), and CD28 (Tonbo Biosciences, San Diego, CA). Cells were then fixed and permeabilized before the addition of anti-Ki67 (BD biosciences, Franklin Lakes, NJ) to assess proliferation. This panel allows for the identification of B cells (CD20) and T cells (CD8b, CD4) as well as naïve and memory subsets as previously described^14^. Another 10^6^ BALF cells were stained with CD206, CD14, HLA-DR, CD8a, CD123, CD16 (Biolegend, San Diego, CA), CD11c (Invitrogen, Waltham, MA), and Granzyme-B (BD Biosciences) to assess frequency of innate immune cells^14^. To measure frequency of antigen-specific T cells, monocyte-derived macrophages, and DCs after MAH infection, 10^6^ BALF cells were stimulated with 1 mg/mL MAH lysate in the presence of 0.013 mg/mL brefeldin A for 16 hrs. At the end of the incubation, cells were surface stained with CD8b (Beckman Coulter), CD4, CD20, CD14, and HLA-DR (Biolegend); cells were then fixed and permeabilized followed by staining with IL-17, IFNγ (Biolegend), and TNF (Invitrogen). Data were acquired using an Attune NxT (Life Technologies, Carlsbad, CA) and analyzed with FlowJo software (TreeStar, Ashland, OR).

### ELISA:

IgG antibody titers against MAH were determined using enzyme-linked immunosorbent assay (ELISA). Plates were coated with 1 mg/mL MAH bacterial lysate overnight at 4°C. The plates were washed three times with 0.05% Tween-PBS. Heat-inactivated (56°C for 30 min) plasma samples were then added in 3-fold dilutions in duplicates for 1.5 h at room temperature. After washing with 0.05% Tween-PBS, Goat anti-monkey IgG (Fc) HRP (Brookwood Biomedical, Jemison, AL) was diluted 1:5000 then added to each well and incubated for 1–1.5 hr. The plates were then washed three times with 0.05% Tween-PBS and incubated for 20 min in a solution containing o-phenylenediamine·2HCl (OPD) substrate (Sigma-Aldrich, St. Louis, MO) diluted in substrate buffer and 30% H_2_O_2_. The reaction was stopped by adding 1M HCl to each well. Absorbance at 490 nm was measured using the SpectraMax iD3 (Molecular Devices, San Jose, CA) plate reader. Each plate included a positive control which was used to normalize the data.

### Luminex:

Levels of immune mediators in BALF supernatant were analyzed using the R&D 36-plex NHP XL Cytokine Premixed Kit (Bio-Techne, Minneapolis, MN). The following analytes were measured: BDNF, CCL2, CCL5, CCL11, CCL20, CD40L, CXCL2, CXCL10, CXCL11, CXCL13, FGF basic, G-CSF, GM-CSF, Granzyme B, IFNα, IFNβ, IFNγ, IL-1β, IL-10, IL-12 p70, IL-13, IL-15, IL-17A, IL-2, IL-21, IL-4, IL-5, IL-6, IL-7, IL-8, PDGF-AA, PDGF-BB, PD-L1, TGFα, TNF, VEGF. Samples were acquired using the MAGPIX xMAP (Luminex Corporation, Austin, TX). Data were analyzed using the Luminex Xponent software with an 8 point logistic regression curve.

#### 16s amplicon sequencing and bioinformatics analysis:

Amplification of the hypervariable V4 region of the 16s rRNA gene was performed using the 515F/806R PCR primers; the forward primers were conjugated with a 12-bp barcode^50^. Each reaction was run in duplicate and prepared with GoTaq master mix (Promega Corporation, Madison, WI) according to manufacturer’s instructions. Cycling conditions were: 94°C for 3 min, 37 cycles of 94°C for 45 s, 50°C for 1 min, and 72°C for 1 min, followed by a final cycle at 72°C for 10 min. The PCR products were multiplexed using Quant-iT PicoGreen dsDNA Assay Kits and dsDNA Reagents (Thermo Fisher). The resulting library was then spiked with ~ 15–20% PhiX and sequenced on an Illumina MiSeq. Raw FASTQ 16s rRNA gene amplicon sequences were processed using the QIIME2 analysis pipeline^51^. Sequences were demultiplexed and filtered using DADA2, resulting in the removal of chimeric sequences and the generation of an amplicon sequence variant (ASV) table^52^. MAFFT was used to align the sequence variants while FastTree2 was utilized to construct a phylogenetic tree^53,54^. Taxonomy was assigned to sequence variants using q2-feature-classifier against the SILVA database (release 138)^55^. The samples were rarified to 10,000 sequences per sample to normalize the sequencing depth between samples. QIIME2 was also used to generate alpha diversity metrics, which include richness (as observed ASV), Shannon evenness, and phylogenetic diversity, while beta diversity was estimated using weighted and unweighted UniFrac distances^56^. The LEfSe algorithm allowed for the identification of differentially abundant bacteria between groups with a linear discriminant analysis score cutoff of 2^57^.

### Statistical analysis:

Statistical analysis was performed using GraphPad Prism software (GraphPad Software Inc., La Jolla, CA) using a one- or two-way ANOVA with the Benjamini and Hochberg false discovery rate (FDR) correction method for multiple comparisons or a two-tailed, unpaired parametric Welch’s or Student’s T-test.

## Supplementary Material

This is a list of supplementary files associated with this preprint. Click to download.

• SUPPLEMENTALFIGURESANDLEGENDS.docx

• SupplementaryTable.docx

## Data Availability

The 16S amplicon sequencing data set supporting the conclusions of this article is available in the Sequence Read Archive (SRA), PRJNA1398294, https://dataview.ncbi.nlm.nih.gov/object/PRJNA1398294?reviewer=ofrrgropc50j4d87leqfb5nrku.
